# LGB (lesbian, gay, and bisexual) state policy protections and substance use disparities

**DOI:** 10.1093/haschl/qxaf029

**Published:** 2025-03-14

**Authors:** Alice Guan, Paul Wesson, David V Glidden, Rita Hamad, Judy Y Tan, Scarlett L Gomez

**Affiliations:** Department of Epidemiology and Biostatistics, University of California, San Francisco, San Francisco, CA 94143, United States; Department of Epidemiology and Biostatistics, University of California, San Francisco, San Francisco, CA 94143, United States; Department of Epidemiology and Biostatistics, University of California, San Francisco, San Francisco, CA 94143, United States; Department of Social and Behavioral Sciences, Harvard School of Public Health, Boston, MA 02115, United States; Department of Biomedical Sciences, Cedars Sinai, West Hollywood, CA 90069, United States; Department of Epidemiology and Biostatistics, University of California, San Francisco, San Francisco, CA 94143, United States

**Keywords:** epidemiology, LGB health, substance use, social policies

## Abstract

LGB (lesbian, gay, and bisexual) individuals have higher rates of tobacco and alcohol use than the general population. While protective social policies have been found to reduce these disparities, their long-term impact remains largely unknown. In this study, we used data from waves 3 (2001–2002) and 4 (2008–2009) of the National Longitudinal Study of Adolescent to Adult Health to assess the impact of exposure to LGB state policy protections during emerging adulthood on substance use in young adulthood. Using multivariable Poisson models, we evaluated whether emerging adulthood was a critical period of exposure and quantified the relative reduction in substance use disparities between LGB and heterosexual individuals living in more protective states. Findings suggest that LGB individuals living in states with more policy protections during emerging adulthood had a significantly lower prevalence of tobacco use and binge drinking in young adulthood compared with those in less protective states. These findings were not observed among heterosexual individuals, indicating that policy effects were specific to LGB individuals. Furthermore, these protections appeared to reduce overall substance use disparities, especially among female participants. It is critical to continue evaluating policy protections to safeguard the health of the LGB community, especially considering the potential erosion of these vital protections.

## Introduction

LGB (lesbian, gay, and bisexual) disparities in tobacco and alcohol use have been well documented.^1-4^ A recent systematic review found that smoking prevalence among LGB individuals ranged from 38% to 59%, significantly higher than the national averages of 28% to 35%.^5^ Additionally, studies using nationally representative datasets suggest that binge drinking and general alcohol use are more common among LGB individuals compared with the general population.^6-8^ Tobacco and alcohol use among adolescents has also been found to vary geographically, with smoking rates ranging from 9.5% in Utah to 27.0% in West Virginia,^9^ and binge drinking rates ranging from 11.0% in Utah to 21.4% in North Dakota.^10^ Given the severe health risks posed by tobacco and alcohol use and their high prevalence among LGB individuals, understanding geographic factors associated with these behaviors is critical to implementing interventions that address these disparities.

Nearly 1 in 6 emerging adults identify as LGBT (lesbian, gay, bisexual, transgender), with state-level percentages of LGBT adults ranging from 4.1% in West Virginia and Mississippi to 14.3% in Washington, DC.^11^ In the United States, LGB individuals face discrimination and victimization,^12^ which elevates their risk of substance use.^13^ Structural interventions, including supportive policies at institutional, local, and national levels, have been found to mitigate these risks. For instance, a meta-analysis found that LGB individuals lacking supportive environments—such as support from parents or other adults or connection to the LGB community—were at higher risk of substance use.^14^ Moreover, states with high structural stigma—such as fewer same-sex households and lacking employment protections—have greater disparities in drug use between LGB and heterosexual individuals.^15^ Conversely, implementing LGB-supportive policies in schools, workplaces, and communities has been shown to reduce drug use, decrease discrimination, and improve well-being for LGB individuals.^16,17^ For instance, 1 study found that LGB residents in states with nondiscrimination policies protecting sexual minorities experienced less minority stress,^18^ a mechanism that has been linked to substance use disparities in this population.^14^ Despite these findings, the American Civil Liberties Union reported that over 500 bills restricting LGBTQ+ (lesbian, gay, bisexual, transgender, and queer or questioning) rights were introduced across the United States in 2024, with many targeting access to health care, education, and other protections for LGBTQ+ individuals.^19^ This surge in anti-LGBTQ+ legislation likely reflects the broader sociopolitical trend of cultural and ideological polarization in the United States as well as reaction to the visibility and legal progress of LGBTQ+ communities. This trend highlights the urgent need for studies examining the impacts of protective policies that safeguard the health of this community.

Recent evidence suggests that LGB policies can have long-term effects on the health and well-being of LGB individuals. For example, a study of gay immigrant men in Sweden found that those exposed to high levels of structural stigma early in life reported lower life satisfaction and poorer mental health in adulthood.^20,21^ However, research on whether early exposure to LGB policy protections in the United States has lasting effects on substance use is limited. Given that emerging adulthood (ages 18–25) is a pivotal developmental phase with major life transitions,^22^ and also crucial milestones in sexual orientation development,^23^ we hypothesized that exposure to LGB policies during this period can have a lasting impact. This study aims to (1) investigate whether exposure to LGB policy protections during emerging adulthood influences substance use in young adulthood and (2) examine whether these policies contribute to reducing substance use disparities between LGB and heterosexual individuals.

## Data and methods

### Sample

We used data from the National Longitudinal Study of Adolescent to Adult Health (Add Health), a large, national, and population-based dataset, which includes longitudinal measurements of sexual orientation and health behaviors. Add Health recruited 20 475 individuals aged 12–19 years (wave 1, 1995–1996) using a multistage, stratified, school-based sampling approach, and followed the cohort into adulthood.^24^ Wave 3 included 15 197 respondents (ages 17–26 years), with measurements taken between 2001 and 2002, and wave 4 included 15 608 respondents (ages 24–32 years), with measurements taken between 2008 and 2009.

#### Defining the LGB sample

The LGB sample was defined based on the question: “Choose the description that best fits how [you think] about [yourself], from the following response options: ‘100% heterosexual (straight),’ ‘mostly heterosexual (straight), but somewhat attracted to people of [their] own sex,’ ‘bisexual—that is, attracted to men and women equally,’ ‘mostly homosexual (gay), but somewhat attracted to people of the opposite sex,’ ‘100% homosexual (gay),’ ‘not sexually attracted to either males or females,’ and ‘don’t know’.” Individuals were characterized as LGB individuals if they indicated they were “bisexual,” “mostly homosexual,” or “100% homosexual.”

### Measures

#### Exposure: LGB state policies

For both wave 3 (2001/2002) and wave 4 (2008/2009) data, we defined a state-level indicator of LGB policy protections, based on previously established methods of examining structural stigma.^15,25-27^ Policies included the following: (1) the inclusion of sexual orientation as a protected category for employment discrimination laws, (2) hate crime provisions protecting individuals based on sexual orientation, (3) legal recognition of same-sex marriage, and (4) the right to same-sex adoption. Participants were categorized as living in states with 0, 1, 2, or 3/4 LGB protections during emerging adulthood. We collapsed categories for states with 3 or 4 protections due to sparse data. Measures reflected the policies in effect during each wave; for instance, if a state had employment discrimination protections in 2002, respondents in that state were classified as exposed to this policy during wave 3.

#### Outcomes: substance use

Current tobacco use was measured with the following question: “During the past 30 days, on how many days did you smoke cigarettes?” Those who smoked on at least 1 day were classified as current smokers. This definition is consistent with the National Survey on Drug Use and Health (NSDUH) definition of current smoking, which has been widely used in public health research.^28^ While other studies may use different thresholds to classify smoking behavior (eg, 25 days or more to define daily smoking), the NSDUH cutoff of at least 1 day provides a standardized measure and also reflects the Healthy People 2030 goals of reducing current cigarette smoking among young people.^29^ Binge drinking was measured with the question, “Over the past 12 months, on how many days did you drink 5 or more drinks in a row?” For females, the threshold was 4 or more drinks. Responses included the following: “none,” “1 or 2 days,” “once a month or less,” “2 or 3 days a month,” and “every day or almost every day.” Since Add Health did not use a standard binge-drinking definition, we created a dichotomous variable indicating whether an individual had at least 1 binge-drinking episode in the past month. This aligns with the US National Institute on Alcohol Abuse and Alcoholism and Healthy People metrics for binge drinking.^30,31^

#### Covariates

We adjusted for sociodemographic characteristics that could confound the relationship between policy exposure and outcomes, including race/ethnicity (White, Black or African American, American Indian or Alaskan Native, Asian or Pacific Islander, Hispanic), biological sex, and age. We additionally adjusted for several census-tract–level contextual variables derived from the Census of Population and Housing Summary Files that might influence the exposure and the outcomes.^32^ These included the proportion of people below the poverty level, those with a college degree, and those in same-sex-partner households. All neighborhood characteristics were treated as continuous variables. We did not adjust for individual-level variables like personal income or education, as these are part of the causal pathway relevant to our research question.

### Statistical analysis

#### Main analysis

We first described the sociodemographic characteristics of our full sample, stratified by sexual orientation. Differences between heterosexual and LGB individuals were assessed using chi-square tests for categorical variables and *t* tests for continuous variables. In addition to presenting *P* values, we computed standardized differences to highlight the magnitude of these differences, which was especially relevant in our large sample where statistical significance might be affected by the volume of data.

We tested 2 primary hypotheses. First, we evaluated whether exposure to LGB policies during the period of emerging adulthood constituted a critical period of exposure for tobacco and alcohol use in young adulthood. This was done using 2 multivariable Poisson models with robust estimators (Figure S1). These models included random intercepts for state and census tract to account for correlations at these levels, and model estimates were exponentiated to provide prevalence ratios. In the first model, we examined the overall association between exposure to LGB protections during emerging adulthood and substance use in young adulthood. In the second model, we investigated whether these associations persisted after accounting for exposure to LGB protections in later adulthood, with a significant result indicating that emerging adulthood was a critical period. We tested these hypotheses separately for LGB individuals and heterosexuals, using the latter as a negative control group, as we anticipated that policy protections would not significantly affect this group.

Second, we evaluated whether more LGB state protections were associated with larger reductions in substance use disparities for LGB individuals. We computed a combined variable of sexual identity and number of LGB state policy protections. Heterosexual individuals residing in states with fewer protections served as the reference category. We estimated prevalence ratios for substance use between LGB and heterosexual individuals using Poisson models with robust variance estimators. Models were adjusted for race/ethnicity, biological sex, and age and tract-level poverty, college education, and same-sex-partner households. We conducted this analysis in the full sample, and also stratified by biological sex.

#### Secondary analysis

Next, we conducted secondary analyses to test the robustness of our findings. First, to address the possibility that the dichotomization of policy protections might oversimplify the complexity of the policy landscape, we repeated our analysis using a categorical variable for LGB policies.

Second, we examined whether substance use disparities varied with different definitions of sexual orientation, following prior work.^33^ While our main analysis relied on self-reported sexual orientation to define the sexual minority sample, sexual behavior and attraction are also critical. Therefore, we tested alternative definitions by incorporating 2 additional questions: one about same-sex romantic attraction and another about same-sex sexual activity. These responses were combined with self-reported sexual orientation and biological sex to refine our LGB sample definition, as detailed in Table S2. Finally, although our primary focus was on the overall policy context, we also explored the impact of each individual policy component on our outcomes of interest.

## Results

### Sample characteristics

Of the 17 764 participants, based on data reported in wave 4, 4.4% identified as LGB (Table 1). In our entire sample, a majority lived in states with less than 3 LGB state protections during both emerging (78.6%) and young (64.7%) adulthood, and a majority (81.6%) lived in the same state in both time periods. A higher proportion of LGB individuals lived in more protective states, reported female biological sex (63.1% compared with 52.7% for heterosexual participants), and lower total personal income (mean: $26.8 K compared with $35.0 K for heterosexual) and college degree completion (27.6% compared with 31.8% for heterosexual participants).

**Table 1.** Sample characteristics for wave 4 respondents to the National Longitudinal Study of Adolescent to Adult Health by sexual minority status.

	Total (*n* = 13 010)	Heterosexual (*n* = 12 393)	LGB (*n* = 617)	*P*	SD
Age, y	28.9, 1.8	28.9, 1.8	28.6, 1.7	<.01	0.16
Female biological sex	7073 (54.4%)	6681 (53.9%)	392 (63.5%)	<.01	−0.20
Total personal income (in 10 000s of USD)	34.7, 37.9	35.1, 38.5	27.2, 22.0	<.01	0.25
Race and ethnicity				.03	−0.09
Asian or Pacific Islander	372 (2.9%)	349 (2.8%)	23 (3.7%)		
Black	2746 (21.1%)	2617 (21.1%)	129 (20.9%)		
Latino	2041 (15.7%)	1920 (15.5%)	121 (19.6%)		
Native American	372 (2.9%)	349 (2.8%)	23 (3.7%)		
White	6961 (53.5%)	6651 (53.7%)	310 (50.2%)		
College degree obtained	4281 (32.9%)	4104 (33.1%)	177 (28.7%)	.02	0.09
Census-tract proportion					
With college degree	0.3, 0.2	0.3, 0.2	0.3, 0.2	.32	−0.04
Same-sex households	0.0, 0.1	0.0, 0.1	0.0, 0.0	.90	0.01
Households less than poverty level	0.2, 0.2	0.1, 0.2	0.2, 0.1	.02	−0.11
Number of LGB state policy protections, wave 3				.47	−0.06
0	4653 (35.8%)	4441 (35.8%)	212 (34.4%)		
1	3502 (26.9%)	3343 (27.0%)	159 (25.8%)		
2	2197 (16.9%)	2094 (16.9%)	103 (16.7%)		
3	2658 (20.4%)	2515 (20.3%)	143 (23.2%)		
4	71 (0.5%)	66 (0.5%)	5 (0.8%)		
Number of LGB state policy protections, wave 4				<.01	−0.12
0	2337 (18.0%)	2244 (18.1%)	93 (15.1%)		
1	4301 (33.1%)	4099 (33.1%)	202 (32.7%)		
2	1738 (13.4%)	1682 (13.6%)	56 (9.1%)		
3	4634 (35.6%)	4368 (35.2%)	266 (43.1%)		
4	802 (6.2%)	754 (6.1%)	48 (7.8%)		
Same state, waves 3 and 4	10 406 (81.5%)	9926 (81.6%)	480 (78.9%)	.09	0.07
Substance use outcomes					
Current smoker	4529 (35.1%)	4245 (34.5%)	284 (46.4%)	<.01	−0.24
Past-year binge drinking	2735 (21.0)	2602 (21.1%)	133 (21.6%)	<.01	−0.10

Values are presented as *n* (%) or mean, SD. Data from wave 3 were collected between 2001 and 2002; data from wave 4 were collected between 2008 and 2009.

Abbreviations: LGB, lesbian, gay, bisexual; SD, standardized difference between groups for both continuous and categorical variables; USD, US dollars.

### Main analysis

#### LGB policies in emerging adulthood and substance use in young adulthood

Results from the analysis of emerging adulthood as a critical period of exposure to LGB policies are presented in Table 2. LGB individuals living in more protective states during emerging adulthood had a lower prevalence of tobacco use (prevalence ratio [PR] = 0.76; 95% CI: 0.65 to 0.90) and binge drinking (PR = 0.69; 95% CI: 0.50 to 0.96) compared with those living in less protective states. This association was slightly attenuated for tobacco use after accounting for exposure to these policies in later adulthood (PR = 0.80; 95% CI: 0.66 to 0.96), but remained statistically significant, providing evidence of emerging adulthood as a critical period of exposure for this outcome. For binge drinking, the association with exposure to policy protections in emerging adulthood was explained largely by exposure to these policies in later adulthood (PR = 0.73; 95% CI: 0.47, 1.13). We did not observe similar associations among the non-stigmatized group (ie, heterosexual individuals), providing evidence for the specificity of these policies for LGB individuals.

**Table 2.** LGB state policy protections and substance use in young adulthood, by sexual orientation: Add Health waves 3 and 4.

	Model 1	Model 2
LGB individuals (*n* = 794)		
Tobacco use	0.76 [0.65, 0.90]*	0.80 [0.66, 0.96]*
Binge drinking	0.69 [0.50, 0.96]*	0.73 [0.47, 1.13]
Heterosexual (*n* = 16 993)		
Tobacco use	0.93 [0.82, 1.04]	0.92 [0.81, 1.03]
Binge drinking	1.03 [0.89, 1.19]	0.99 [0.85, 1.16]

Values are prevalence ratios [95% CI]. *P < .05. Model 1 represents the overall association between exposure to LGB state policy protections in emerging adulthood and substance use outcomes. Model 2 represents the remaining association, after accounting for exposure to LGB state policy protections in later life. All models adjusted for the following variables: age, biological sex, race/ethnicity, and tract-level proportions of people who have obtained a college degree, same-sex unmarried partners, and people living under the poverty level. State policy variables were measured in wave 3, and outcomes in wave 4. Emerging adulthood was defined as ages 18–26 years and young adulthood was defined as ages 24–32 years, corresponding to Add Health participants in waves 3 and 4.

Abbreviations: Add Health, National Longitudinal Study of Adolescent to Adult Health; LGB, lesbian, gay, bisexual.

#### LGB state protections and substance use disparities

Next, we evaluated whether exposure to more policy protections led to significant reductions in substance use disparities among LGB individuals (Table 3). LGB individuals living in states with fewer protections had a higher prevalence of both tobacco use (PR = 1.43; 95% CI: 1.29 to 1.58) and binge drinking (PR = 1.36; 95% CI: 1.19 to 1.55) compared with heterosexual people living in states with fewer protections. However, LGB individuals living in more protective states had a 14% reduction in tobacco use disparity (PR = 1.23; 95% CI: 1.01 to 1.49) and a complete reduction in binge-drinking disparity (PR = 0.99; 95% CI: 0.73 to 1.34). In analysis stratified by biological sex, this pattern was primarily observed among female participants.

**Table 3.** Substance use disparities between LGB and heterosexual individuals by exposure to LGB state policy protections in emerging adulthood: Add Health waves 3 and 4.

	Tobacco use		Binge drinking	
	0–2 LGB policy protections	3+ LGB policy protections	0–2 LGB policy protections	3+ LGB policy protections
Full sample				
Heterosexual	Ref	0.91 [0.81, 1.03]	Ref	1.04 [0.89, 1.21]
LGB	1.43 [1.29, 1.58]*	1.23 [1.01, 1.49]*	1.36 [1.19, 1.55]*	0.99 [0.73, 1.34]
Female				
Heterosexual	Ref	0.85 [0.69, 1.04]	Ref	1.17 [0.96, 1.42]
LGB	1.60 [1.37, 1.87]*	1.34 [1.06, 1.70]*	1.68 [1.41, 2.02]*	1.60 [1.00, 2.50]
Male				
Heterosexual	Ref	0.94 [0.87, 1.02]	Ref	0.98 [0.85, 1.13]
LGB	1.13 [0.96, 1.32]	1.06 [0.87, 1.29]	1.07 [0.87, 1.32]	0.46 [0.32, 0.66]*

Values are prevalence ratios [95% CI]. *P < .05. Estimates were derived from Poisson models with robust variance estimators, and adjusted for age, sex (for full-sample analysis), race, and tract-level proportion of people who (1) lived below the poverty level, (2) have completed a college degree, and (3) were in same-sex-partner households. The sample included Add Health participants in wave 4 who also had data on LGB policies available during emerging adulthood (wave 3). State policy variables were measured in Wave 3, and outcomes in wave 4.

Abbreviations: Add Health, National Longitudinal Study of Adolescent to Adult Health; LGB, lesbian, gay, bisexual; Ref, reference.

**Figure 1.**
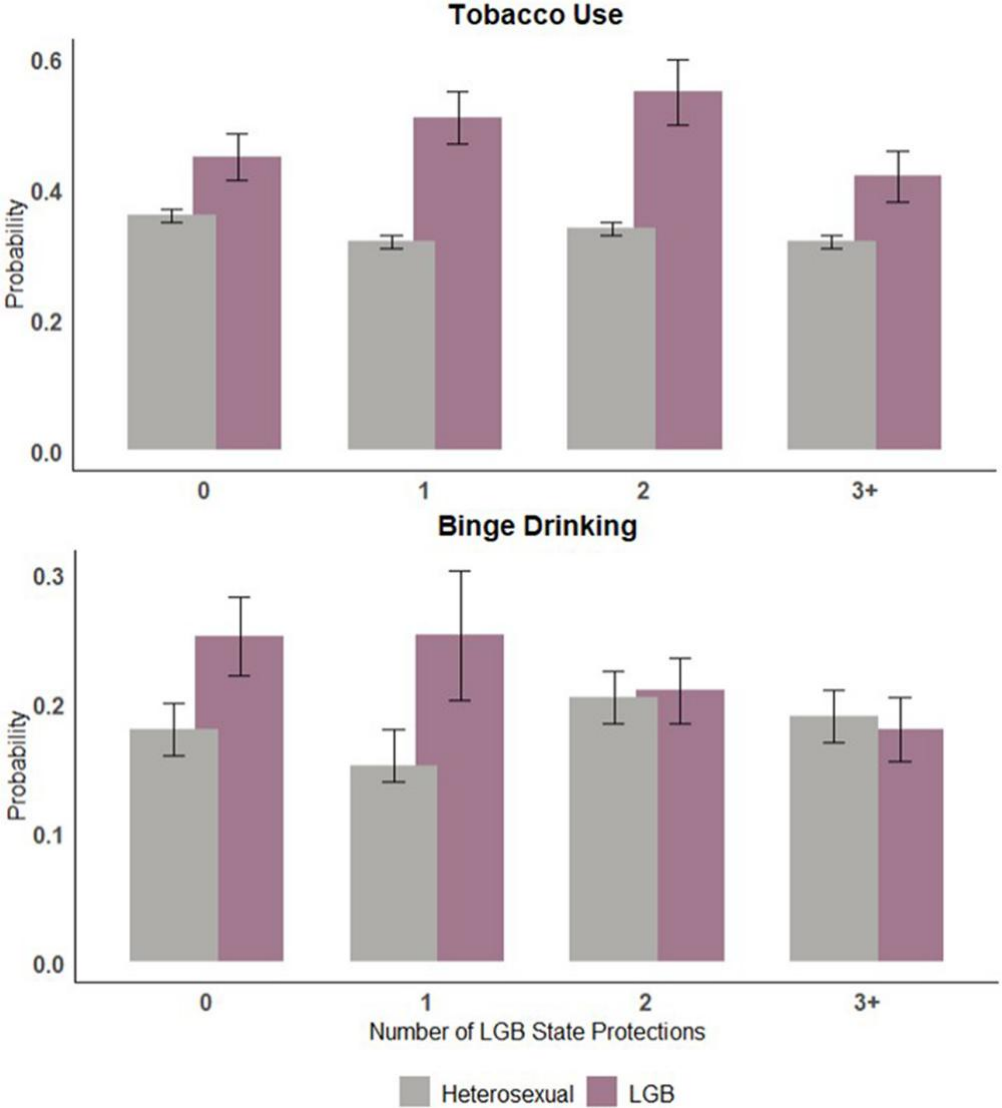
Model-based predicted probabilities of substance use based on the number of LGB state protections. The plots represent predicted probabilities, which were estimated from Poisson models with robust estimators that included an interaction between sexual orientation and number of LGB state policy protections as a categorical variable. Models were adjusted for age, sex (for full-sample analysis), race, and tract-level proportion of people who (1) lived below the poverty level, (2) have completed a college degree, and (3) were in same-sex-partner households. Error bars are based on the standard error. When sample sizes are large (as is the case in this analysis), SEs and 95% CIs are roughly equivalent, and the use of SEs was selected to simplify presentation. The sample included Add Health participants in wave 4 who also had data on LGB policies available during emerging adulthood (wave 3). State policy variables were measured in wave 3, and outcomes in wave 4. Abbreviations: Add Health, National Longitudinal Study of Adolescent to Adult Health; LGB, lesbian, gay, bisexual.

For heterosexual individuals, we observed no difference in the prevalence of tobacco use or binge drinking based on the number of state policy protections, offering additional evidence to support the specificity of these policy protections. These observed differences in the relationship between state policies and sexual orientation were significant in interaction analysis (Figure 1).

### Secondary analysis

Results from analysis treating the number of LGB policy protections as a categorical variable are presented in Table S1. For LGB individuals, we did not observe meaningful differences in the prevalence of tobacco use between states with 0, 1, or 2 policies. However, LGB individuals living in states with 3 or more policies had a lower prevalence of tobacco use compared with those living in states with no protections (PR = 0.81; 95% CI: 0.66 to 0.99). Although there seemed to be a trend of decreasing prevalence of binge drinking among LGB individuals with an increasing number of policies, these estimates had CIs that encompassed null effects.

Results from analyses using various definitions of our sexual minority sample are presented in Table S2. With regard to tobacco use, we observed a consistent pattern of lower relative disparity between LGB and heterosexual individuals in more protective states. However, for binge drinking, while we observed a lower relative disparity for LGB individuals residing in states with more protective policies, a complete elimination of disparity was only observed for the most restrictive definition of LGB. As we expanded our definition of the sexual minority population to include sexual behaviors and attraction, the observed disparity was reduced but not eliminated.

Associations between each independent policy component and substance use are presented in Table S3. For LGB individuals, employment discrimination policies were associated with a reduction in both tobacco use (PR = 0.82; 95% CI: 0.69 to 0.96) and binge drinking (PR = 0.63; 95% CI: 0.46 to 0.86). Marriage equality policies were similarly protective toward tobacco use (PR = 0.81; 95% CI: 0.70 to 0.95) and binge drinking (PR = 0.64; 95% CI: 0.45 to 0.90). We observed no association between hate crime statutes and same-sex adoption policies with either outcome.

## Discussion

This study contributes to the growing body of research that state policy protections are beneficial for the health of the LGB community. We found that exposure to LGB state protections during emerging adulthood was associated with lasting effects on tobacco use behaviors in young adulthood. These protections may help reduce exposure to minority stress, including stigma, discrimination, and structural inequities, which are well-documented drivers of substance use as a coping mechanism among LGB individuals.^14,34,35^ Anti-LGBT social environments can exacerbate these stressors, and further tobacco and alcohol use.^26,36,37^

Our finding suggests that the period of emerging adulthood presents a window of opportunity for policies to shield against the impacts of discrimination experienced by LGB individuals, and can lead to reductions in long-term substance use. This is critically important as early-onset tobacco use is known to predict longer smoking histories, higher daily cigarette consumption, and habitual daily smoking,^38,39^ all of which increase lifetime risk of chronic conditions.^40^ While school-based programs (eg, programs that promote inclusive social climates) have emerged in recent years to support LGB youth during childhood and adolescence,^41-44^ there is a crucial need to extend these efforts into emerging adulthood.

Additionally, our study suggests that living in states with more LGB state protections can minimize disparities in tobacco use and binge drinking, especially for LGB women. One possible explanation is that LGB women, compared with LGB men, face compounded stressors tied to both gender and sexual orientation, including greater exposure to workplace discrimination, social exclusion, and economic insecurity, which may heighten their vulnerability to coping mechanisms such as tobacco and alcohol use. Protective state policies may specifically benefit LGB women by addressing some of these systemic inequities. Additionally, national data show that baseline disparities in tobacco and alcohol use are more pronounced among women than men,^45^ suggesting that LGB women may have more to gain from policies that mitigate these disparities. Future research should further explore how the unique intersection of gender and sexual orientation shapes the effectiveness of state protections.

Compared with less protective states, in states with more LGB protections, tobacco use disparities were reduced and binge-drinking disparities were eliminated. Notably, we observed a threshold effect regarding the number of protective policies needed to impact long-term substance use. States with 0, 1, or 2 policies showed minimal differences in tobacco use, while states with 3 or more policies saw notable reductions in disparities. This suggests that the impact of protective policies may not be linear and could require reaching a certain level of protections before significant effects are observed. While this study primarily focused on the overall policy environment, secondary analyses indicated that employment discrimination protections and same-sex marriage policies were more strongly associated with reductions in tobacco use and binge drinking among LGB people. These findings suggest that addressing multiple domains of life—such as finances (employment discrimination protections), interpersonal relationships (hate crime provisions), and family (same-sex marriage and adoption policies)—may work synergistically to create environments that mitigate stigma and minority stress. Future research should utilize new resources, such as those from the Movement Advancement Project, to further investigate how more recent policies (and their combinations) and their implementation affect long-term outcomes for LGB communities. It is also crucial to recognize that policy implementation and enforcement can vary widely across states, and each state's unique policy mix might influence outcomes differently. Therefore, while our findings suggest that more protective environments are associated with reduced disparities in tobacco and alcohol use, caution is needed before making definitive causal claims.

This study has several limitations. Our analysis used restricted data files that did not specify the states in which participants resided, limiting our ability to assess whether other state-specific policies or factors might have influenced the relationship between LGB policies and substance use outcomes. We partially mitigated this issue by adjusting for area-level factors associated with substance use, such as educational attainment. The substance use data were self-reported, which may lead to underreporting. For instance, a recent study found that 15.7% of young adults underreported tobacco use.^46^ However, since exposure to LGB policies and substance use outcomes were measured independently (via geocoded state of residence and survey responses, respectively), any bias from self-reporting is likely nondifferential and may have attenuated the estimates towards the null. Relatedly, our study defines current smoking following the NSDUH definition, which does not differentiate between daily, occasional, and experimental tobacco use. Thus, our classification of “current smokers” includes individuals with different smoking patterns, and could potentially bias our estimates of smoking prevalence and its association with LGB state protections. Notably, the most likely direction of this bias would be an underestimation of disparities in tobacco use behaviors between LGB and heterosexual people, as evidence suggests that sexual minorities are more likely to be daily smokers, whereas heterosexual young-adult smokers have a higher proportion of occasional and experimental use.^47,48^ By collapsing all smokers into a single category, our study may dilute the true difference in smoking prevalence, leading to more conservative estimates of disparities and possibly underestimating the protective effects of state policies. Additionally, in this study we were unable to examine associations within racial/ethnic subgroups. While our models adjusted for race/ethnicity as a covariate, our sample sizes for certain racial/ethnic subgroups within the LGB population were too small to support stratified analyses. Additionally, our data did not include state policies affecting transgender individuals, an area that has seen significant political and social changes in recent years. Future research should include policies specifically impacting transgender individuals to provide a more comprehensive understanding of policy effects on health. Last, there is a potential for selection bias due to attrition between the initial cohort assembly and the data waves used in this study.

Over the past few decades, significant progress has been made in advancing LGB equality, including landmark achievements like the 2015 Obergefell vs Hodges decision on marriage equality and state-specific bans on conversion therapy. However, there have also been notable setbacks. In 2024, the American Civil Liberties Union (ACLU) reported tracking over 500 anti-LGBTQ bills across the United States.^19^ Given our findings on the long-term impact of policies on LGB health and their role in reducing disparities, the potential reversal or weakening of existing protections is concerning. It is essential for policymakers and researchers to remain engaged in efforts that safeguard the rights and well-being of LGB individuals.

## Supplementary Material

qxaf029_Supplementary_Data
