# Supplementary material for: LGB (lesbian, gay, and bisexual) state policy protections and substance use disparities
Source: Health Aff Sch. 2025 Mar 14;3(3):qxaf029. doi: 10.1093/haschl/qxaf029 (PMC11907768; doi:10.1093/haschl/qxaf029)
Supplement: qxaf029_Supplementary_Data [file qxaf029_supplementary_data.zip › HASCHOLAR_supplementary_materials.pdf]

SUPPLEMENTAL MATERIALS

LGB state protections and sexual minority substance use disparities

Contents:

Supplemental Methods.
- Life course model specifications
- Brief description of LGB policies

Supplemental Figure 1. Illustrative depictions of the study hypotheses and model specifications

Supplemental Table 1. Substance use disparities by exposure to number of state policies, Add Health waves 3 and 4

Supplemental Table 2. Substance use disparities by exposure to LGB state policies using different criteria to define sexual minority sample, Add Health waves 3 and 4

Supplemental Table 3. Associations between each LGB state policy and substance use outcomes

Life course model specifications

We first evaluated whether there was an overall association between exposure to LGB state policy protections in emerging adulthood (measured in Add Health wave 3) and substance use (measured in Add Health wave 4). To do this, we implemented the following model:

$$\log\left(\mathrm{E}\left(\mathrm{Y}_{\mathrm{ij}}\right)\right) = \beta_0 + \beta 1 * W3LGBPolicies_{ij} + \beta 2 * IindividualCovariates_{ij} + \beta 4 * CensusTractCovariates_j + u_j + v_k + \varepsilon_{ij}$$

where:

$\log\left(\mathrm{E}\left(\mathrm{Y}_{\mathrm{ij}}\right)\right)$ is the log expected value of Wave 4 outcome for individual i, in census tract j and state k

β0 is the intercept

β1 represents the total effect of exposure to LGB policies at W3 on substance use in W4

β2 and β3 are vectors of individual and census tract level covariates

u_j is the random intercept at the tract level (w4_tract).

v_k is the random intercept at the state level (w4_state).

ε_{ij} is the error term representing the residual variability.

In the above equation, β1 was the estimate reported in Table 2 (model 1), and intended to represent the association illustrated with the blue arrow above.

Second, we evaluated whether exposure to LGB state policy protections explained the association that we observed in the first stage of modeling. In other words, we added a variable representing a participant's exposure to LGB state policy protections in Wave 4, as summarized by the below equation:

$$\log\left(\mathrm{E}\left(\mathrm{Y}_{\mathrm{ij}}\right)\right) = \alpha_0 + \alpha 1 * W3LGBPolicies_{ij} + \alpha 2 * W4LGBPolicies_{ij} + \alpha 3 * IindividualCovariates_{ij} + \alpha 4 * CensusTractCovariates_j + u_j + v_k + \varepsilon_{ij}$$

where:

$\log\left(\mathrm{E}\left(\mathrm{Y}_{\mathrm{ij}}\right)\right)$ is the log expected value of Wave 4 outcome for individual i, in census tract j and state k

α0 is the intercept

α1 represents the total effect of exposure to LGB policies at W3 on substance use in W4

α2 represents the remaining direct effect of exposure to LGB policies at W3 on substance use at W4 accounting for W4 LGB policy exposure

α3 and α4 are vectors of individual and census tract level covariates

u_j is the random intercept at the tract level (w4_tract).

v_k is the random intercept at the state level (w4_state).

ε_{ij} is the error term representing the residual variability.

In the above equation, α1 was the estimate reported in Table 2 (model 2), and intended to represent the association illustrated with the blue arrow above.

Brief description of LGB policies in Wave 3

The percentage of participants who lived in a state with the following LGB policies were as follows:

Employment discrimination protections	4502 (30.24%)
Hate crime statutes	9108 (61.17%)
Allowed same sex marriage	2847 (19.12%)
Allowed same sex adoption	2680 (18.00%)

A majority of participants lived in states with no LGB state policy protections. Below is a distribution of the specific policies by the total number of state protections:

N policy protections	N participants
Zero	5261
One	4026
Hate Crime Provision Only	3842
Employment Discrimination Only	184
Two	2627
Hate Crime Statutes + Same-Sex Adoption	1569
Employment Discrimination + Hate Crime	437
Employment Discrimination + Same-Sex Adoption	426
Employment Discrimination + Same-Sex Marriage	195
Three	3183
Employment Discrimination + Hate Crime + Same-Sex Marriage	2575
Employment Discrimination + Hate Crime + Same-Sex Adoption	608
Four	77

Supplemental Figure 1. Illustrative depictions of the study hypotheses and model specifications

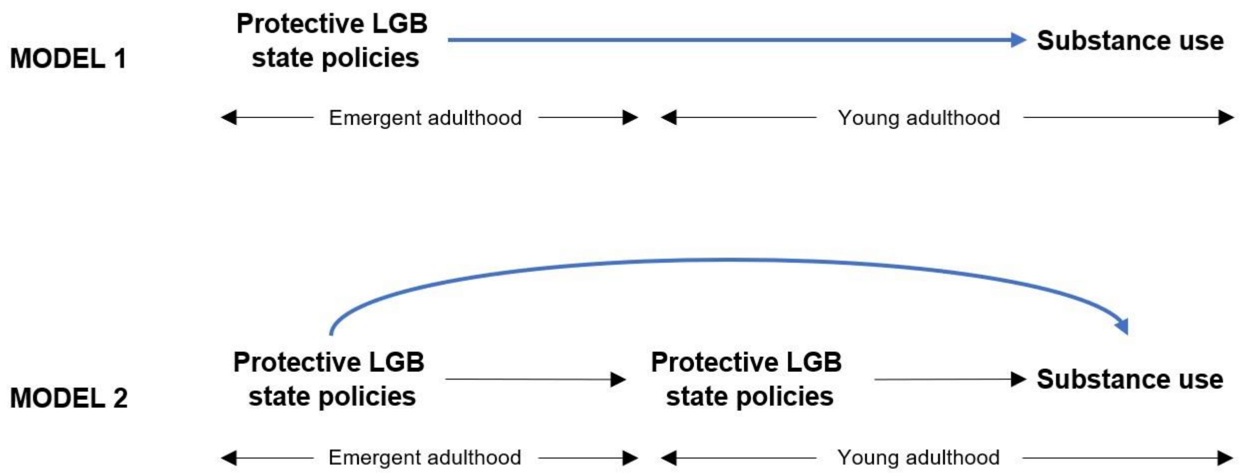

Supplemental Table 1. Substance use disparities by exposure to number of state policies, Add Health waves 3 and 4

	Tobacco Use		Binge Drinking	
	**LGB**	**Heterosexual**	**LGB**	**Heterosexual**
	Prevalence Ratio [95% Confidence Interval]			
**Number of LGB policies**				
Zero	Ref	Ref	Ref	Ref
One	1.07<br>[0.86, 1.35]	0.88 *<br>[0.79, 0.98]	1.10<br>[0.74, 1.64]	0.84<br>[0.67, 1.06]
Two	1.14<br>[0.91, 1.43]	0.93<br>[0.82, 1.06]	0.89<br>[0.64, 1.25]	1.08<br>[0.90, 1.30]
Three or more	0.81*<br>[0.66, 0.99]	0.88<br>[0.77, 1.00]	0.70<br>[0.48, 1.00]	1.02<br>[0.87, 1.18]

*p<0.05, Estimates shown in the table above were derived from Poisson models with robust variance estimators, and adjusted for age, sex (for full sample analysis), race, and tract-level proportion of people who a) lived below the poverty level, b) have completed a college degree, and c) were in same-sex partner households. The sample included Add Health participants in Wave 4 who also had data on LGB policies available during emerging adulthood (Wave 3). State policy variables was measured in Wave 3, and outcomes in wave 4.

Supplemental Table 2. Substance use disparities by exposure to LGB state policies using different criteria to define sexual minority sample, Add Health waves 3 and 4

	**0-2 SM policy protections**	**3+ SM policy protections**
Prevalence Ratio [95% CI] of tobacco use for LGB (vs. heterosexual)		
LGB Definition 1	1.42 [1.29, 1.57]*	1.36 [1.16, 1.58]*
LGB Definition 2	1.58 [1.45, 1.73]*	1.56 [1.30, 1.86]*
LGB Definition 3	1.56 [1.45, 1.68]*	1.42 [1.23, 1.64]*
LGB Definition 4	1.51 [1.42, 1.61]*	1.43 [1.31, 1.56]*
LGB Definition 5	1.51 [1.42, 1.61]*	1.41 [1.30, 1.54]*
Prevalence Ratio [95% CI] of binge drinking for LGB (vs. heterosexual)		
LGB Definition 1	1.36 [1.19, 1.56]*	0.92 [0.74, 1.15]
LGB Definition 2	1.48 [1.32, 1.66]*	1.08 [0.81, 1.44]
LGB Definition 3	1.44 [1.31, 1.58]*	1.16 [1.05, 1.29]*
LGB Definition 4	1.50 [1.38, 1.64]*	1.14 [1.03, 1.25]*
LGB Definition 5	1.49 [1.37, 1.62]*	1.12 [1.03, 1.23]*

*p<0.05; LGB Definitions are as follows: (1) Individuals identified as "bisexual," "mostly homosexual," or 100% homosexual. (2) Those falling under Definition 1 + those who reported engaging in sexual activity with someone of the same sex. (3) Those falling under Definition 2 + those who have ever reported experiencing same-sex romantic attraction. (4) Those falling under Definition 3 + those who identified as "mostly heterosexual." (5) Those falling under Definition 4 + those who responded with "don't know" when queried about their sexual orientation.

Supplemental Table 3. Associations between each LGB state policy and substance use outcomes

	LGB	Heterosexual
Prevalence Ratio [95% CI] of tobacco use		
Employment discrimination protections	0.82 [0.69, 0.96]*	0.95 [0.86, 1.05]
Hate crime statutes	1.03 [0.87, 1.22]	0.87 [0.79, 0.95]*
Allow same-sex marriage	0.81 [0.70, 0.95]*	0.97 [0.87, 1.08]
Allow same-sex adoption	0.99 [0.81, 1.22]	1.00 [0.89, 1.11]
Prevalence Ratio [95% CI] of binge drinking		
Employment discrimination protections	0.63 [0.46, 0.86]*	1.11 [0.94, 1.31]
Hate crime statutes	0.99 [0.72, 1.36]	0.93 [0.81, 1.07]
Allow same-sex marriage	0.64 [0.45, 0.90]*	1.04 [0.86, 1.25]
Allow same-sex adoption	0.90 [0.67, 1.21]	0.99 [0.85, 1.16]

*p<0.05; Prevalence ratios and confidence intervals shown in the table above were derived from Poisson models with robust variance estimators, and adjusted for age, sex (for full sample analysis), race, and tract-level proportion of people who a) lived below the poverty level, b) have completed a college degree, and c) were in same-sex partner households. The sample included Add Health participants in Wave 4 who also had data on LGB policies available during emerging adulthood (Wave 3). Each state policy variable was measured in Wave 3, and outcomes in wave 4.
